# Functional MRI in Prostate Cancer Detection

**DOI:** 10.1155/2014/590638

**Published:** 2014-07-23

**Authors:** Sandeep Sankineni, Murat Osman, Peter L. Choyke

**Affiliations:** Molecular Imaging Program, National Cancer Institute, NIH, 10 Center Drive, MSC 1182, Building 10, Room B3B69, Bethesda, MD 20892-1088, USA

## Abstract

Multiparametric magnetic resonance imaging (MP-MRI) has emerged as a promising method for the detection of prostate cancer. The functional MRI components of the MP-MRI consist of the diffusion weighted MRI, dynamic contrast enhanced MRI, and magnetic resonance spectroscopic imaging. The purpose of this paper is to review the existing literature about the use of functional MRI in prostate cancer detection.

## 1. Introduction

In 2014, it is estimated that 233,000 men in the United States will be diagnosed with prostate cancer, and 29,480 men will ultimately die as a result [1]. To date, most men are diagnosed based on a rising serum prostate specific antigen (PSA) or positive digital rectal examination (DRE) followed by a 12-core template biopsy. This strategy often overdiagnoses low grade posterior lesions and underdiagnoses higher grade anterior lesions of the prostate [2, 3]. Multiparametric MRI (MP-MRI) has recently emerged as a valuable tool for determining risk of prostate cancer. Directed biopsy of the prostate based on the MRI decreases both overdiagnosis and underdiagnosis of anterior cancer lesions [4]. In this review we discuss the key components of a state-of-the-art MP-MRI in the diagnosis and management of prostate cancer.

MP-MRI can be performed at 1.5 T or 3.0 T with or without an endorectal coil (ERC). The highest signal to noise ratio is achieved at 3 T with an ERC but acceptable results can be achieved at 3 T without an ERC. Scans at 1.5 T with an ERC are generally acceptable and good results can still be achieved at 1.5 T without an ERC provided that specific equipment features (newer hardware/coils are generally required) are critical to success. The literature is currently limited regarding the use of ERC versus non-ERC. However, recently a comparison study was done by Turkbey et al. to investigate ERC versus non-ERC at 3 T. This study was done in 20 patients with whole-mount histopathology confirmation. It was determined that the sensitivity of MP-MRI increased by 31%, and the positive predictive value was 16% higher. The ERC MP-MRI was also able to lower the mean lesion size at detection by 21% [5]. Larger, multi-institutional studies will need to be completed to ultimately conclude on ERC versus non-ERC at 1.5 T and 3 T.

**Figure 1:**
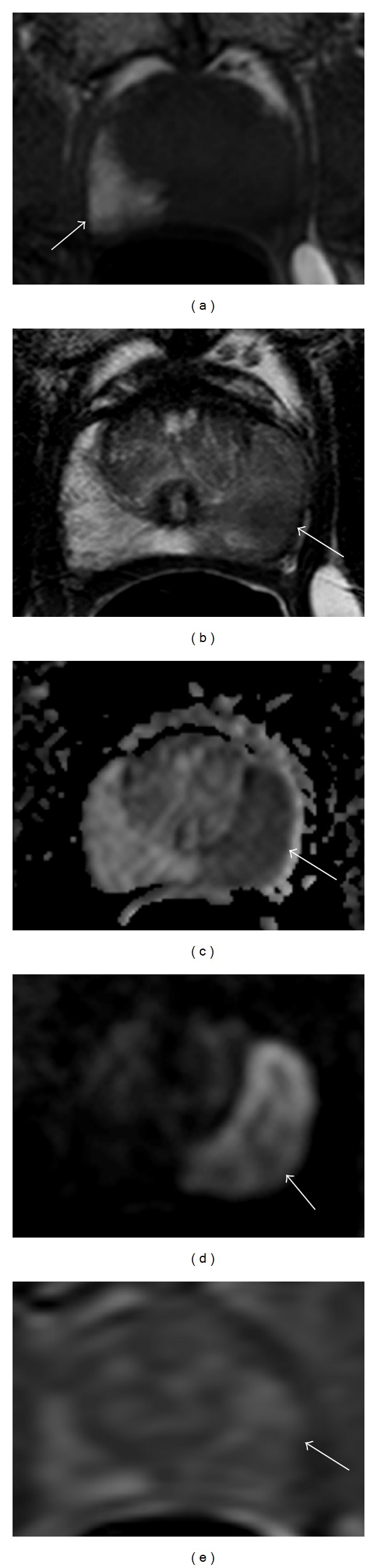
A 57-year-old patient, with PSA of 5.80, Gleason 4 + 5 on multiple cores, undergoing postbiopsy staging MP-MRI. Axial T1 weighted MRI shows biopsy related residual hemorrhage in right midperipheral zone (arrow) (a). Axial T2 weighted MRI shows a large left midperipheral zone lesion (b). Axial diffusion weighted MRI showing a large lesion extending throughout the left midperipheral zone (arrow) (c). High “*b*” value DWI (*b* = 2000) showing strong enhancement of the left midperipheral zone lesion (arrow) (d). Axial dynamic contrast enhanced imaging showing early enhancement in the left midperipheral zone (arrow) (e).

**Figure 2:**
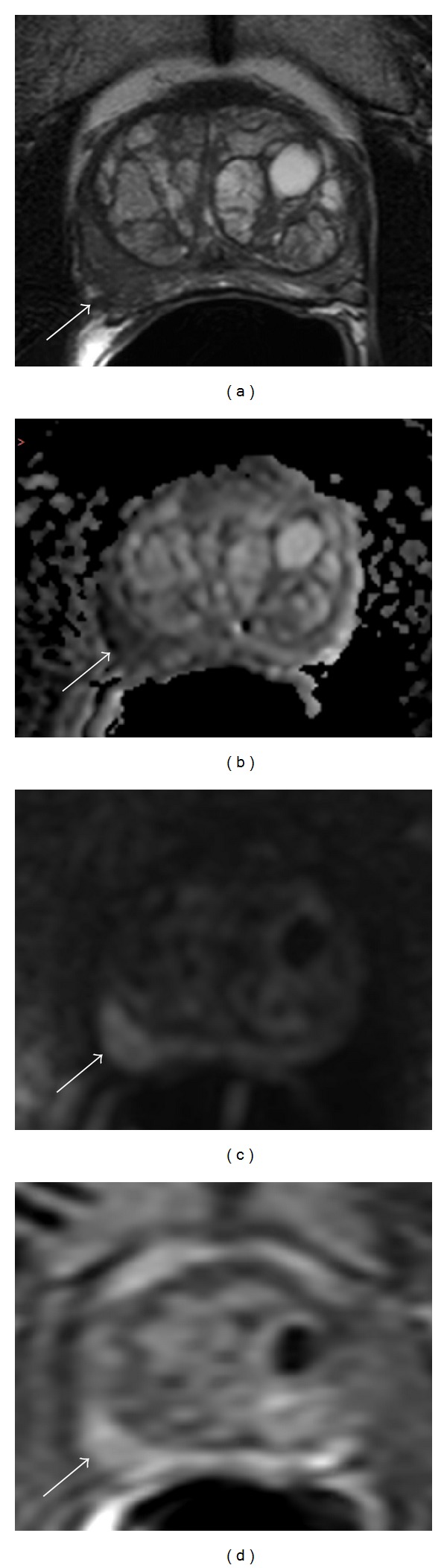
A 58-year-old patient with a PSA of 4.84, previously diagnosed with Gleason 3 + 4 cancer on 12-core template biopsy. Axial T2 weighted image showing hypointense signal in right midperipheral zone with signs of capsular irregularity and suspicion of extracapsular extension (ECE) (arrow) (a). Axial diffusion weighted image showing hypointense area in the right midperipheral zone (arrow) (b). Axial high “*b*” value DWI (*b* = 2000) hyperintense in the right midperipheral zone (arrow) (c). Axial dynamic contrast enhanced image showing strong early enhancement in right midperipheral zone (arrow) (d). Patient underwent an MP-MRI followed by a MRI/transrectal ultrasound (TRUS) fusion guided biopsy upgrading to a Gleason 4 + 3 in all three MP-MRI determined targets of the right midperipheral zone.

T1 and T2 weighted (T1W and T2W) images of the prostate are the main tools for the primary visualization of zonal and anatomical features [6]. T1W images are obtained primarily to rule out biopsy related residual hemorrhage, which can diminish accuracy of prostate MRI as hemorrhage can easily mimic prostate cancer. Patients who have undergone prostate biopsy should wait 8–12 weeks to clear any hemorrhage prior to an MRI. If substantial hemorrhage is still present, then the patient should be rescheduled for a later date (Figures 1(a)–1(d)). T2W images provide excellent soft tissue contrast and spatial resolution and, therefore, full evaluation of prostate and its relation to other anatomical features such as the urethra, bladder wall, capsule, seminal vesicles, and neurovascular bundles. In the clinical setting, these structures should be routinely visualized for a proper evaluation. The determination of extracapsular extension (ECE) depends on clear visualization of the external prostate capsule and the identification of any tumor transgressing the capsule. Additionally, the seminal vesicles should be well evaluated to exclude a possible invasion. For an ideal seminal vesicle evaluation, they need to be sufficiently distended; therefore, patients should refrain from ejaculation for three days prior to the MRI, which is helpful in this regard. Thus, T1W MRI is critical for hemorrhage and T2W MRI is critical in providing anatomic detail as well as suggesting the location and extent of cancer which is generally seen as low signal foci on the T2W MRI (Figure 2(a)).

## 2. Diffusion Weighted Imaging

Diffusion weighted magnetic resonance imaging (DW MRI) reflects the diffusion of water protons within tissue. Signal decreases on DW MRI in proportion to the restriction of motion of water. This reflects cellular density, and other obstructions to the free flow of water such as extracellular matrix [7]. DW-MRI is acquired by sequentially applying multiple magnetic field gradients, known as “*b*” values, to calculate apparent diffusion coefficient (ADC) values and construct ADC maps [8]. Lower diffusion coefficients (e.g., cancer) are seen as low signal intensity focus on ADC maps but as high signal intensity focus on high *b* value images (Figures 1(e) and 2(b)). Normal glandular prostate tissue allows unrestricted free water movement and displays isointense regions on ADC maps [9]. Since cancers express increased cellular density, water diffusion within the extracellular space of cancerous tissue is often restricted [10]. Therefore, on raw DW MRI, tumors appear hyperintense compared to background due to the restricted water diffusion, whereas, on ADC maps, cancers are generally characterized by their lower signal and thus appear hypointense on ADC maps more than that of normal tissue [11] (Figure 2(c)). The lower the ADC value is, the more likely the prostate lesion is malignant and of higher grade. Thus, DW MRI provides important insights into the microscopic nature of prostate tissue and is commonly used as a fast and effective clinical tool for the detection of prostate tumors [12].

ADC values in malignant tumors are also lower than normal tissues in the transitional zone (TZ) and correspond to increased signal on DW MRI when compared to the background signal [12–14]. BPH causes the TZ of the prostate to become heterogeneous in signal intensity pattern depending on the degree in which the stromal or glandular tissues change [15]. This heterogeneity makes evaluation of prostate lesions more difficult in the TZ as tumors and BPH nodules can have overlapping signal pattern on ADC maps [16]. For this reason, ADC maps are mostly used in conjunction with T2W MR images to delineate, well-circumscribed benign prostatic hyperplasia (BPH) nodules from more poorly defined tumor lesions in the TZ.

Combined anatomic T2W MRI and functional DW MRI have been reported to improve cancer detection in several studies. In a study by Sato et al., tumor foci with a diameter > 4 mm and Gleason scores > 6 were evaluated with T2W MRI and DW MRI (high *b* value = 600 s/mm^2^) at 1.5 T. The area under the receiver operating characteristic curve (AUC) using the combined approach (T2W and DW MRI) was 0.89 in comparison to T2W imaging alone, which had an AUC of 0.81 [17]. Similarly, in another study by Morgan et al., sensitivity and specificity for T2W versus T2W/DW MRI were evaluated. It was shown that with the addition of the DW MRI sequence, sensitivity increased from 50% to 79.6% and specificity increased from 73.2% to 80.8% (*P* < 0.001) [18]. ADC values derived from DW MRI have also been reported to inversely predict Gleason scores; low ADC values are associated with higher grade tumor foci [19, 20]. For instance, in a study of 48 patients with visible tumors, Turkbey et al. reported a negative correlation between tumor ADC values and their corresponding Gleason scores [19]. In another study by Hambrock et al., prostate lesions with the highest restriction determined on ADC maps were associated with higher grade tumors [21].

The addition of DW MRI to standard imaging protocols has improved diagnostic accuracy of MRI and is relatively quick to obtain. Recently, higher *b* values (>1000 s/mm^2^) have been suggested to offer higher tumor visibility and detection. Rosenkrantz et al. compared *b* values of 1000 s/mm^2^ and 2000 s/mm^2^ in 29 patients and compared tumor to peripheral zone (PZ) contrast with both* b* values. They found that a *b* value of 2000 s/mm^2^ resulted in improved tumor sensitivity (*P* < 0.001) and contrast (*P* = 0.067) since normal or benign PZ tissue showed very low signal on high *b* value images whereas neoplastic tissue remained bright [22]. However, high *b* value DW MRI produced noisier images with lower SNR values [23]. Lower “high *b*” value imaging produces higher signal and so intermediate *b* values of 1400–1500 s/mm^2^ have been suggested as reasonable alternatives. The high signal requirements are particularly problematic for nonendorectal coil imaging, although good high *b* value can be achieved without the ERC. Rectal bowel gas can cause distortions on DW MRI that can interfere with the diagnostic value of this sequence [24].

In addition to standard ADC values, other measures of ADC distribution have been evaluated such as diffusion kurtosis (DK). Kurtosis is useful as an indicator of the “peakedness” of a distribution. Metens et al. evaluated ADC values using diffusion kurtosis and found that ADC values of cancer foci were significantly lower than that of benign PZ. Specifically, ADC values were significantly (*P* < 0.001) lower in prostate cancer lesions (0.79 ± 0.14 *μ*m^2^/ms) compared to benign PZ (1.23 ± 0.19 *μ*m^2^/ms) [25]. Unlike standard DW imaging models which assume water displacement is similar to that of free diffusion, the DK model quantifies the degree to which water diffusion is non-Gaussian and thus has the ability to better reflect the complex structure of the tissue.

With regard to prostate cancer staging, DW MRI is limited in depicting extracapsular extension due to its lower spatial resolution. However, DW MRI has been reported to be effective for the detection of seminal vesicle invasion (SVI) [26]. A study by Kim et al. showed that the area under the receiver operation characteristic curve (AUC) was greater (0.897) for a dual T2W and DW MRI approach as opposed to T2W images (0.779) alone for detection of SVI [27]. Further studies are being done to evaluate the role of DW MRI for structures surrounding the prostate gland such as urinary bladder carcinomas [28]. DW MRI has potential to support other clinical and laboratory biomarkers and has important clinical implications in predicting tumor aggressiveness, selecting appropriate therapies, and improving the accuracy of biopsies for detected tumors.

## 3. Dynamic Contrast Enhanced MRI

Dynamic contrast enhanced (DCE) MRI has the ability to track the enhancement of tissue and therefore provide a measure of angiogenesis, on which tumor growth is dependent [29]. DCE is performed by acquiring fast T1W images, prior to, during, and after contrast injection. DCE-MRI detects the arrival and uptake of gadolinium based contrast agents before they wash out and is related to tumor vascularity and permeability. The MR contrast agent (i.e., gadolinium chelate) must be given as a bolus by an intravenous route often with an automated injector, making it relatively more “invasive” and requiring more resources than other MP-MRI sequences (T2W and DWI).

In DCE MRI, tumors are highlighted by their strong enhancement compared to the normal background tissue (Figure 2(d)). Tumor enhancement appears earlier and washes out more quickly in comparison to normal tissue. This reflects the process of angiogenesis—whereby new vessels are formed with higher vascular permeability to deliver more nutrients to the growing tumor, thus aiding in its growth [30–33]. DCE has high sensitivity, reported to be in the range of 74–96%, which is useful for the preliminary detection of tumors [33]. It is widely used for staging and in monitoring therapeutic response of prostate cancer. A study by Hara et al. found the sensitivity and specificity of DCE to be 73% and 88%, respectively, among a group of 50 patients [34]. Another study by Ocak et al. determined that a combination of T2W MRI with DCE MRI increased cancer detection sensitivity in the PZ by 16% over T2W MRI alone. However, this high sensitivity can also lead to an increased false positive rate, and in this study the specificity of detection in the PZ dropped from 98% to 92% [35]. In the transitional zone, BPH nodules strongly enhance but do not wash out as quickly as prostate tumors [36, 37]. Therefore, outside of well-defined BPH nodules, DCE MRI can be helpful in defining TZ cancers as well.

There are several methods of analyzing DCE data which increase in complexity and the need for specific software. Generally speaking there are three methods for interpreting these images: qualitative, semiquantitative, and quantitative. Qualitative evaluation, the easiest and the most popular approach, involves the visual detection of focal early, strong enhancement with early washout—compared with that of normal tissue [38]. Naturally, while this method is simple, it is also subject to observer bias and often requires considerable experience. Semiquantitative analysis is an assessment of the time-signal curve and includes measurement of AUC, time to peak enhancement, and initial slope. Dynamic curves are classified as persistent (Type 1), plateau (Type 2), and decline after initial slope (Type 3). The latter, Type 3, is frequently associated with prostate cancer—although a combination of curve types is actually the most common. In quantitative evaluation, pharmacokinetic modeling is performed using two compartment models that determine *K* ^trans⁡^ (forward volume transfer constant) and *k*_ep_ (reverse reflux rate constant between extracellular space and plasma) rate constants [34]. As a result of the variability in evaluation methods the value of DCE is still in debate. DCE MRI has limited value in local staging; however it was reported by Turkbey et al. that the accuracy of DCE for the detection of capsular extension, SVI, and neurovascular bundle involvement was 84%, 97%, and 97%, respectively [39].

## 4. Magnetic Resonance Spectroscopic Imaging

**Figure 3:**
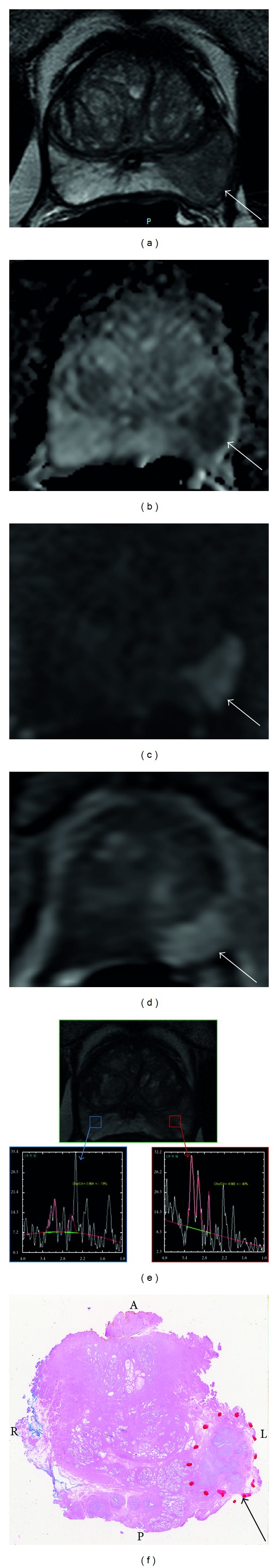
A 53-year-old patient with a PSA of 13.59, previously diagnosed with Gleason 3 + 3 cancer on 12-core template biopsy. Axial T2 weighted image shows a large hypointense signal in left midperipheral zone with capsular bulge (arrow) (a). Axial diffusion weighted imaging showing left hypointense signal in left midperipheral zone (arrow) (b). Axial high “*b*” value DW MRI (*b* = 2000) with hyperintense signal in left midperipheral zone (arrow) (c). Axial dynamic contrast enhanced imaging showing strong early enhancement in the left midperipheral zone (arrow) (d). Magnetic resonance spectroscopic imaging showing an increased choline : citrate ratio in the left mid peripheral zone (outlined in red). Normal choline : citrate ratio is seen in the contralateral peripheral zone (outlined in blue) (e). Patient underwent a MP-MRI followed by MRI/TRUS fusion guided biopsy, at which time the patient was upgraded to a Gleason 4 + 4. Prostatectomy was then performed and whole-mount pathology confirms Gleason 4 + 4 in 30% of the left lobe (f).

Among the sequences which comprise the MP-MRI, proton magnetic resonance spectroscopic imaging (MRSI) is the least frequently used and is mostly limited to the research setting. MRSI provides information about specific metabolites within prostatic tissue. The analysis is performed by measuring the resonance peaks of various biochemical metabolite levels—such as citrate, creatine, and choline. Normal prostate tissue contains an abundant supply of zinc which inhibits aconitase and produces high levels of citrate. Citrate exhibits a unique peak on MR spectroscopy. On the other hand, in prostate cancer downregulation of the ZIP zinc transporters causes a decrease in zinc levels [40]. This reduction in zinc decreases citrate levels by inducing oxidation [41]. Choline levels correlate with cell turnover, as seen in prostate cancer. Thus, as cancers arise, citrate is expected to decline while choline is expected to rise. This ratio of choline to citrate is therefore an indicator of malignancy [42–46] (Figure 3(a)–3(f)).

While MRSI is, in theory, a promising imaging sequence, it requires additional software expertise, training, and support and increases the overall MP-MRI scan time. In a multi-institutional study, organized by the American College of Radiology Imaging Network (ACRIN), it was determined that MR imaging alone was just as effective as MR imaging with MRSI and did not improve tumor localization in the PZ, where most cancers occur. Also, out of the 110 patients in the final study group, only 50% were considered to have achieved good or excellent spectral quality notwithstanding the fact that the study was largely performed in excellent academic centers [47]. For these reasons, MRSI has yet to become widely accepted in standard clinical practice; as a result, research has also slowed down with regard to MRSI. In a recent study of active surveillance in low-risk prostate cancer by Weinreb et al., it was determined that only T2W and DWI were independent predictors of biopsy upgrade [48]. Spectroscopy was therefore not contributory. This study supports the argument against the routine use of MRSI in clinical practice and raises question about the future of MRSI as a component of MP-MRI.

## 5. Further Applications of Functional MRI in Prostate Cancer

In addition to its diagnostic use, functional MP-MRI holds a multipurpose role in the staging, surveillance, and therapy monitoring of prostate cancer.

It is generally accepted that the MP-MRI has a strong negative predictive value for what is considered* clinically significant* prostate cancer, Gleason 4 + 3 and above [49]. In case of rising PSA and negative 12-core template biopsy, a MP-MRI may be acquired to confirm the benign state of the prostate. For a patient with elevated risk (i.e., familial predisposition), with elevated and/or rising PSA and a previous positive 12-core template biopsy of* clinically insignificant* cancer (Gleason 3 + 4 and below), a MP-MRI may be done to confirm the absence of any missed large anterior lesions.

In case of biopsy confirmed, low-risk, organ-confined disease, a patient may decide to take the path of active surveillance. In this case, it may be considered acceptable to schedule a yearly MP-MRI of the prostate—this is in addition to the routine PSA and digital rectal exam screening [50]. Any changes noticed in T2W or functional MP-MRI sequences would be evaluated at that time. The patient could then either continue with active surveillance, avoiding unnecessary subsequent biopsies, as long as the MP-MRI remains stable in the interval, or proceed with follow-up targeted biopsy and treatment.

An important feature of the MP-MRI is its ability to assist in staging of prostate cancer [51]. Since the five-year survival rate is nearly 100% for patients with localized and regional prostate cancer, yet dropping to around 28% in case of distant metastases [52], it is important to have a means of accurate staging so that appropriate adequate treatment can be offered.

Once therapy is started, regardless of the method chosen, effective monitoring will be required to determine disease status. Postfocal therapy PSA may be an unreliable biomarker, and functional MRI may be useful to assist in early recurrence detection, which may signal the need for further investigation.

## 6. Conclusion

When used along with the PSA screening and digital rectal exam, multiparametric MRI is gaining acceptance as a standard of care for the diagnosis and characterization of prostate cancer. The European Society of Urogenital Radiology (ESUR) guidelines for MR prostate are expected to be updated later this year [53]; however, the MP-MRI of prostate should be standardized and follow the minimum MP-MRI protocol as discussed in the ESUR guidelines of 2012 [54]. This includes acquiring a T2W MRI and at least two functional MRI techniques.

Widespread implementation of this diagnostic imaging has raised the need to optimize the various sequences and to further develop novel sequences. Functional MRI, with DW MRI, DCE MRI, and MRSI, is powerful addition to T2W MRI, yet there is still a need for large multi-institutional studies to standardize the evaluation of the MP-MRI
